# *Streptococcus suis* and Porcine Reproductive and Respiratory Syndrome, Vietnam

**DOI:** 10.3201/eid1902.120470

**Published:** 2013-02

**Authors:** Ngo Thi Hoa, Tran Thi Bich Chieu, Sam Do Dung, Ngo Thanh Long, Thai Quoc Hieu, Nguyen Tien Luc, Pham Thanh Nhuong, Vu Thi Lan Huong, Dao Tuyet Trinh, Heiman F.L. Wertheim, Nguyen Van Kinh, James I. Campbell, Jeremy Farrar, Nguyen Van Vinh Chau, Stephen Baker, Juliet E. Bryant

**Affiliations:** Author affiliations: Oxford University Clinical Research Unit, Ho Chi Minh City, Vietnam (N.T. Hoa, T.T.B. Chieu, S.D. Dung, J.I. Campbell, J. Farrar, S. Baker, J.E. Bryant);; Regional Animal Health Office No. 6, Ho Chi Minh City (N.T. Long);; Sub-Department of Animal Health, Tien Giang, Vietnam (T.Q. Hieu);; Sub-Department of Animal Health, Soc Trang, Vietnam (N.T. Luc);; Sub-Department of Animal Health, Thai Binh, Vietnam (P.T. Nhuong);; Oxford University Clinical Research Unit, Hanoi, Vietnam (V.T.L. Huong, H.F.L. Wertheim);; National Hospital for Tropical Diseases, Hanoi (D.T. Trinh, N.V. Kinh,);; Nuffield Department of Clinical Medicine, Oxford, UK (N.T. Hoa, H.F.L. Wertheim, J.I. Campbell, J. Farrar, S. Baker, J.E. Bryant);; Hospital for Tropical Diseases, Ho Chi Minh City (N.V.V. Chau)

**Keywords:** Human cases, porcine reproductive and respiratory syndrome, Streptococcus suis, swine, Vietnam, zoonoses, bacteria, viruses, streptococci

**To the Editor:** *Streptococcus suis*, an opportunistic pathogen of swine, is an emerging zoonotic pathogen among humans (*1*). In Vietnam, *S. suis* is the leading cause of human acute bacterial meningitis (*2*). Infection in humans is associated with direct exposure to infected pigs or infected raw or undercooked pork products (*3*). Of the 35 *S. suis* serotypes, only a limited number are pathogenic for pigs, and clinical cases in humans have most frequently been attributed to serotype 2 (SS2) (*1*). In Vietnam during September 2006–November 2007, the carrier rate of *S. suis* among slaughterhouse pigs was 41% (222/542); SS2 was the most frequently identified serotype in 14% (45/317) of *S. suis* isolations (*4*).

Porcine respiratory and reproductive syndrome (PRRS) is a major disease affecting the swine industry globally; the severity of PRRS in pigs can be increased by co-infection with *S. suis* (*5*). In 2010, PRRS outbreaks in swine were reported in 49 of 63 Vietnamese provinces (Technical Appendix Figure) (*6*). To understand the potential implications of swine PRRS outbreaks for human *S. suis* disease, we investigated co-infections of *S. suis* and PRRS virus (PRRSV) in sick pigs in 3 provinces of Vietnam during the PRRS outbreaks in 2010 (Technical Appendix Figure).

We sampled 108 farms reporting pigs that had a clinical syndrome consistent with PRRSV infections in the provinces of Thai Binh (May), Tien Giang (July), and Soc Trang (July). Samples were blood from sick febrile pigs and postmortem tissue from freshly culled pigs. To confirm swine PRRS outbreaks, we performed reverse transcription real-time PCR on 1 randomly selected plasma sample from each farm (*7*). A total of 103 (95%) plasma samples from 103 farms tested positive for PRRSV (Chinese genotype). We additionally selected 3 PRRSV-positive farms per province for comprehensive PRRSV screening of all 42 sampled pigs; 100% of samples from the 9 farms were PRRSV positive. After swine outbreaks ended, blood samples from 52 healthy pigs from 10 farms that had no recent history of PRRS were collected from Tien Giang Province (March 2011). None of the 52 plasma samples from the 10 control farms tested positive for PRRSV.

We investigated the presence of SS2 in blood and tissue samples from pigs on PRRS- and non-PRRS–affected farms by bacterial culture (Technical Appendix Table). A total of 534 specimens from sick pigs yielded 9 (1.7%) SS2 isolates. One (2%) of 52 specimens from the healthy control pigs yielded a non-SS2 *S. suis* isolate. *S. suis* has been proposed to contribute to the spread of antimicrobial resistance genes to other human pathogenic streptococci (*8*). The antimicrobial susceptibility results of 9 SS2 isolates by disk diffusion (*9*) revealed a high prevalence (6/9, 66%) of resistance to tetracycline, tobramycin, enrofloxacin, and either marbofloxacin or chloramphenicol.

PCR amplification of the *16SrDNA* gene (*10*) and the *cps2J* gene (*2*) was performed on all blood samples to detect *S. suis* and SS2, respectively. Ninety-two (18%) of 521 sick pigs from PRRSV outbreak farms were systemically infected with *S. suis*. In contrast, no healthy pigs from control farms were positive for *S. suis* by PCR (Technical Appendix Table). The SS2-*cps2J*–specific PCR was positive for 58 (11%) of 521 samples, and the *S. suis-16SrDNA* PCR was positive for 55 (11%). Twenty-one of the *16SrDNA*-positive samples also were positive for *cps2J-*PCR, which indicated that 34 (7%) sick pigs were infected with non-SS2 strains. Therefore, SS2 accounted for most (58 [63%] of 92) *S. suis*–positive detections. The bacterial load of SS2 in blood ranged from 1 × 10^3^ CFU/mL^–1^ to 8.3 × 10^6^ CFU/mL^–1^ (median 9.2 × 10^3^ CFU/mL^–1^). Overall, SS2 was found in 58 (11%) sick pigs and on 33 (32%) PRRS outbreak farms. The higher prevalence (92 [18%]) of systemic infections of *S. suis* and SS2 with high bacterial load in pigs from PRRS outbreak farms compared with prevalence on nonoutbreak farms (1 [2%] of 52) suggests increased systemic *S. suis* infections during swine PRRS outbreaks (p = 0.001, Fisher exact test).

We investigated the possible association between swine PRRS outbreaks and human *S. suis* infection. Case reports of confirmed human infections during 2007–2010 at the 2 tertiary referral hospitals in Hanoi and Ho Chi Minh City were reviewed. The number of human *S. suis* infection cases increased in August 2010 in southern Vietnam and doubled in northern Vietnam during May–August and October–November 2010 (Figure). Swine PRRS outbreaks were reported during June–September and March–December 2010 in southern and northern provinces, respectively (*6*) (Technical Appendix Figure). Most patients with *S. suis* infection during these periods resided in provinces reporting swine PRRS outbreaks. Our data suggest a possible temporal association between swine PRRS outbreaks and human *S. suis* infections.

**Figure:**
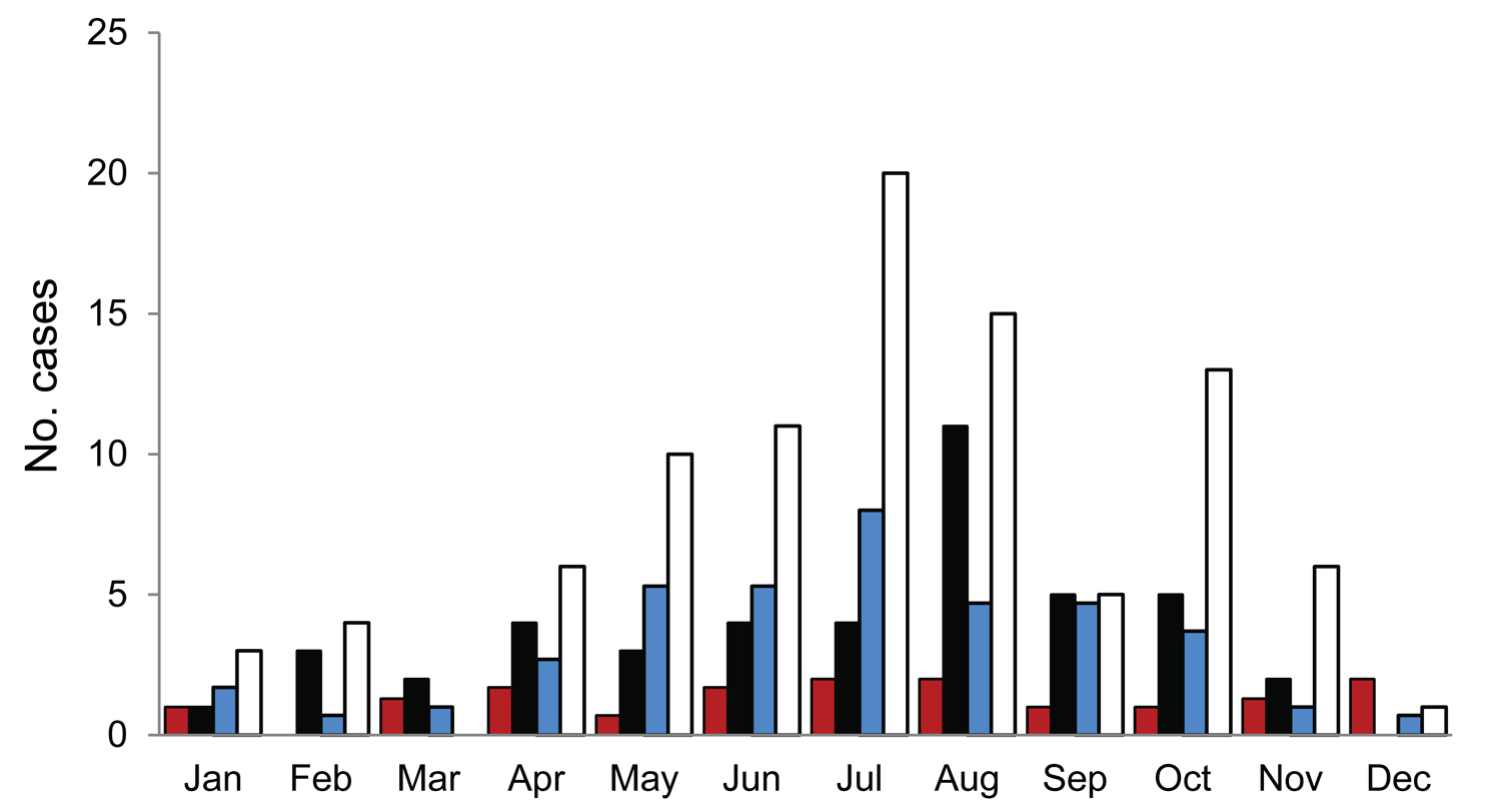
Monthly distribution of human *Streptococcus suis* infections in 2 referral hospitals, Vietnam, 2007–2010. Humans infected with *S. suis* during 2007–2009 are presented as mean total cases per month. Dark gray and black bars represent the number of *S. suis* case-patients at the Hospital for Tropical Diseases in Ho Chi Minh City during 2007–2009 and 2010, respectively. Light gray and white bars represent human *S. suis* cases at the National Hospital for Tropical Diseases in Hanoi during 2007–2009 and 2010, respectively.

We demonstrated increased prevalence of systemic *S. suis* and SS2 infection in pigs co-infected with PRRSV during the 2010 swine outbreaks in Vietnam. The results indicate an increased risk for potential zoonotic transmission of *S. suis* to humans during outbreaks of PRRS in swine.

Technical Appendix Systemic *Streptococcus suis* infections in sick pigs from farms with confirmed porcine reproductive and respiratory syndrome and temporal distribution of outbreaks of porcine reproductive and respiratory syndrome in swine, 3 provinces, Vietnam, 2010.
