# Supplementary material for: Streptococcus suis and Porcine Reproductive and Respiratory Syndrome, Vietnam
Source: Emerg Infect Dis. 2013 Feb;19(2):331–3. doi: 10.3201/eid1902.120470 (PMC3559037; doi:10.3201/eid1902.120470)
Supplement: Technical Appendix — Systemic Streptococcus suis infections in sick pigs from farms with confirmed porcine reproductive and respiratory syndrome and temporal distribution of outbreaks of porcine reproductive and respiratory syndrome in swine, 3 provinces, Vietnam, 2010. [file 12-0470-Techapp-s1.pdf]

Article DOI: http://dx.doi.org/10.3201/eid1902.120470

Streptococcus suis and Porcine Reproductive and Respiratory Syndrome, Vietnam

Technical Appendix

Technical Appendix Table. Systemic *Streptococcus suis* infections in sick pigs from farms with confirmed PRRS in 3 provinces, Vietnam, 2010

Province	No. PRRS outbreak farms*	No. pigs on farms	No. sick pigs reported	No. blood samples†	No. tissue sample sets‡	No. bacteria culture positive	No. *S. suis* culture positive	No. (%) *cps2J* RT-PCR (SS2) positive	No. (%) *16SrDNA* PCR (*S. suis*) positive	No. (%) non-SS2 *S .suis* positive§	Total no. (%) *S. suis* positive¶
Thai Binh	12	530	351	34	0	0	0	32 (94.1)	3 (8.8)	0	32 (94.1)
Soc Trang	41	1,055	927	232	3	9	4	11 (4.7)	15 (6.5)	9 (3.9)	20 (8.6)
Tien Giang	50	2,006	1,715	255	10	8	5	15 (5.9)	37 (14.5)	25 (9.8)	40 (15.7)
Total	103	3,591	2,993	521	13	17	9	58 (11.1)	55 (10.5)	34 (6.5)	92 (17.7)

*PRRS outbreak farms were confirmed by 1) sick pigs with a clinical syndrome consistent with PRRSV infection in farms located in provinces reported for PRRS outbreaks and 2) positive reverse transcription real-time PCR reaction to detect for the presence of *nsp2* gene from PRRS virus in 1 randomly selected blood sample/farm.
†Blood was collected from febrile pigs only. PRRS, porcine reproductive and respiratory syndrome.
‡Tissue sets comprised lung, pulmonary lymph node, liver, spleen and tonsils.
§Samples positive for 16SrDNA but negative for cps2J.
¶Combined cps2J or/and 16SrDNA positive samples.

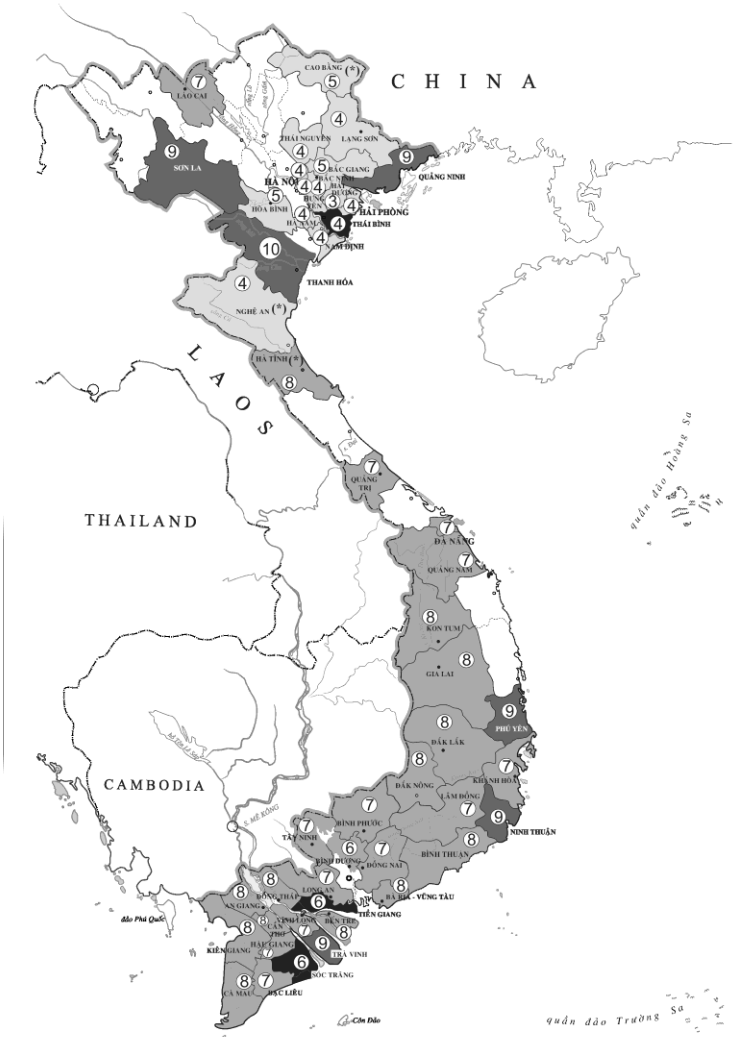

Technical Appendix Figure. Temporal distribution of swine PRRS outbreaks across provinces, Vietnam, 2010. Samples from sick pigs were collected in Thai Binh (northern province), Soc Trang and Tien Giang (southern provinces) (black). Swine PRRS outbreaks across provinces were reported in Vietnam in 2010 (*1*). The month that swine PRRS outbreaks were first reported in each province are indicated by the enclosed number, e.g., March = (3), April = (4).

Reference

1. Tien NN. Situation of blue ear pig diseases in Vietnam and the outbreak control activities [in Vietnamese]. Veterinary Sciences and Techniques. 2011;18:12–20.
